# ﻿A new species of *Trychosis* Förster (Hymenoptera, Ichneumonidae, Cryptinae), with a key to the species known from China

**DOI:** 10.3897/zookeys.1167.104105

**Published:** 2023-06-14

**Authors:** Ying-Jie Ren, Kai Wang, Chun-Lai Zhou, Xing-Bo Cui, Guang-Xin Wang, Man-Hong Liu, Sheng-xin Ye, Zhao-Jun Meng

**Affiliations:** 1 Key Laboratory of Sustainable Forest Ecosystem Management-Ministry of Education, School of Forestry, Northeast Forestry University, Harbin 150040, Heilongjiang, China Northeast Forestry University Harbin China; 2 Hongxinglong Branch, Heilongjiang Naolihe National Nature Reserve Administration, Shuangyashan 155100, Heilongjiang, China Heilongjiang Naolihe National Nature Reserve Administration Shuangyashan China; 3 Natural Resources Rights Survey and Monitoring Institute of Heilongjiang Province, Harbin 150080, Heilongjiang, China Natural Resources Rights Survey and Monitoring Institute of Heilongjiang Province Harbin China

**Keywords:** Agrothereutina, Cryptini, new taxon, taxonomy, wetland

## Abstract

A new species of Ichneumonidae, *Trychosisnaolihense* Meng & Ren, **sp. nov.**, is described and illustrated. Specimens were collected from Naolihe National Natural Reserve, Heilongjiang Province, China. A key to the currently known species from China is provided.

## ﻿Introduction

The genus *Trychosis* Förster, 1869, belonging to the tribe Cryptini of the subfamily Cryptinae (Hymenoptera, Ichneumonidae), currently contains 44 described species (Yu et al. 2016). Of these 44 species, one is recorded from the Oriental region (Cameron 1906), 15 from the Eastern Palaearctic region (of which six also occur in the Western Palaearctic region) (Telenga 1930; Uchida 1930; Barahoei et al. 2015; Yu et al. 2016), 14 from the Western Palaearctic region (van Rossem 1966, 1971, 1990; Schwarz 1994), 20 from the Nearctic region (Townes and Townes 1962), and two from the Neotropical region (Townes and Townes 1962, 1966).

The Western Palaearctic species of *Trychosis* were revised by van Rossem (1966, 1971, 1990). Townes and Townes (1962) studied the Nearctic species and provided a key to the known species from this region. Prior to this study, there was neither a key nor a revision of the Eastern Palaearctic species, only three species of *Trychosis*, *T.indigna* (Kokujev, 1909), *T.legator* (Thunberg, 1822), and *T.yezoensis* (Uchida, 1930), were known from China (Kokujev 1909; Sheng and Sun 2014; Sheng et al. 2016).

Since 2021, a survey to the insect resources has been conducted in the Naolihe National Natural Reserve, Heilongjiang, which aims to track and document the biodiversity of this area. During this survey, a new species of *Trychosis* was collected and discovered in Naolihe Reserve. The primary purpose of this study is to describe and illustrate this new species and provide a key to the currently known species from China.

## ﻿Materials and methods

Specimens were collected using sweeping nets from emerged plants at the Hongqiling farm (46°51′26"N, 133°1′50"E) in Naolihe National Natural Reserve, Heilongjiang. The predominant plant families in this area are Cyperaceae and Gramineae, with *Carexpseudocuraica* and *Calamagrostisangustifolia* as dominant species.

Morphological terminology mostly follows Broad et al. (2018). The type specimen of the new species is deposited in the Center for Biological Disaster Prevention and Control, National Forestry and Grassland Administration (CBDPC). The description and measurements were made under a Leica M205A stereomicroscope with LAS Montage MultiFocus.

## ﻿Results

### ﻿ Trychosis

Taxon classificationAnimaliaHymenopteraIchneumonidae

Förster, 1869

24690845-40E7-5FD0-84AF-99C2F769139A

Trychosis Förster, 1869. Verhandlungen des Naturhistorischen Vereins der Preussischen Rheinlande und Westfalens 25(1868): 187. Type species: Cryptusambigua Tschek.

#### Diagnosis.

Fore wing length 3.2–8.8 mm. Clypeus moderately to strongly convex, with anterior margin almost truncate to strongly convex forward, without tooth. Mesoscutum very short, with dense punctures. Epomia usually present and strong. Epicnemial carina reaching center of mesopleuron close to its anterior margin. Propodeum strongly oblique, anterior and posterior transverse carinae present. Propodeal spiracle almost circular. Areolet usually parallel laterally. Hind wing with vein 1-cu as long as or longer than cu-a. Tergite 1 long, 0.5–0.6× as long as mesosoma. Ovipositor thin, compressed, with nodus.

### ﻿Key to species known from China (females)

**Notes.***Trychosisindigna* (Kokujev, 1909) is not included in the key because only one male specimen is known. It is distinguished from other species in the key by the following combination of characters: complete posterior transverse carina of propodeum; frons with median carina; infumated wings; posterior portion of tergite 1–3 entirely brown.

**Table:** 

1	Clypeus with dense transverse wrinkles. Areolet distinctly convergent anteriorly. Frons almost entirely smooth, shiny. Tergites 6–7 largely white dorsally. Basal 0.7 of hind femur red	***T.naolihense* Meng & Ren, sp. nov.**
–	Clypeus with punctures. Areolet parallel-sided laterally. Frons with punctures or wrinkles. Tergites 6–7 without white spot. Hind femur black or mainly black	**2**
2	Flagellomeres 6–10 white. All tergites and hind femur black	***T.yezoensis* (Uchida, 1930)**
–	Flagellomeres entirely black. Tergites 2–4 red-brown. Hind femur blackish brown	***T.legator* (Thunberg, 1822)**

### ﻿ Trychosis naolihense

Taxon classificationAnimaliaHymenopteraIchneumonidae

Meng & Ren sp. nov.

6BBF1988-8B65-53F7-9170-3A0667C54C42

https://zoobank.org/BCB544DF-A2BB-4C80-A578-D697139C3F3F

Figs 1 , 2–5 , 7–12

#### Type material.

***Holotype*** ♀, China, Heilongjiang Province, National Natural Reserve, Naolihe wetland, Hongqiling farm; 46°51′26"N, 133°1′50"E, 78.9 m elev.; 25 July 2021; leg. Ying-Jie Ren (CBDPC).

#### Diagnosis (female).

Clypeus (Fig. 2) distinctly long, 1.2× as wide as long, with dense, weak, transverse wrinkles. Areolet pentagonal, convergent anteriorly, 3rs-m shorter than 2rs-m. Head, mesosoma, and tergites 1–5 entirely black, tergites 6–7 yellowish white medially and black laterally.

#### Description.

**Figure 1.**
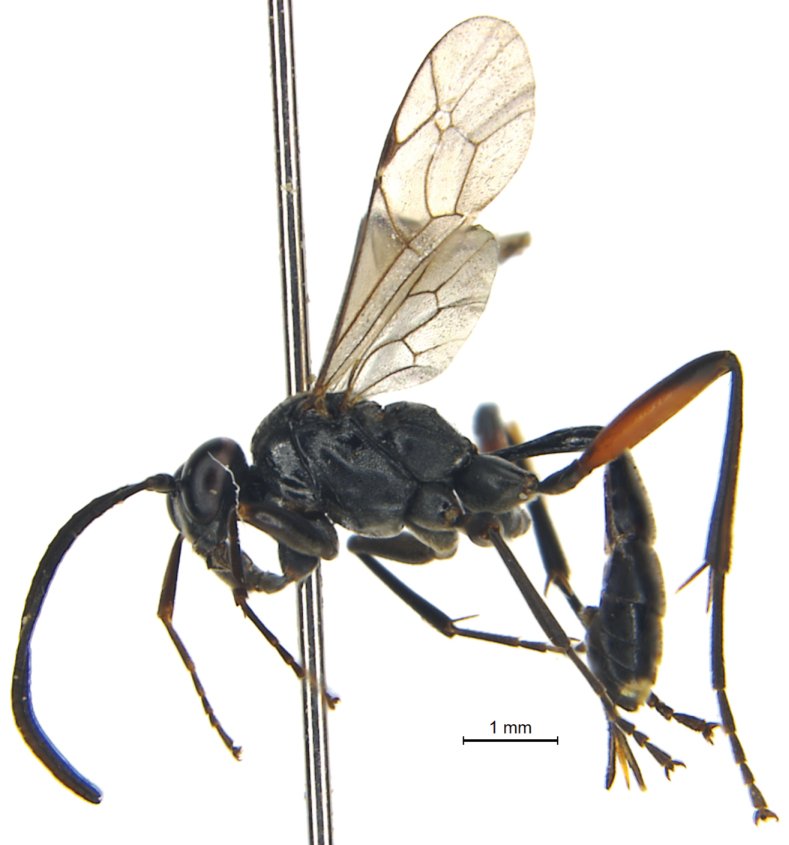
*Trychosisnaolihense* Meng & Ren. Holotype, female, habitus, lateral view.

**Female** (Fig. 1). Body length 7.8 mm. Fore wing length 5.2 mm. Ovipositor sheath length 1.3 mm.

**Figures 2–6.**
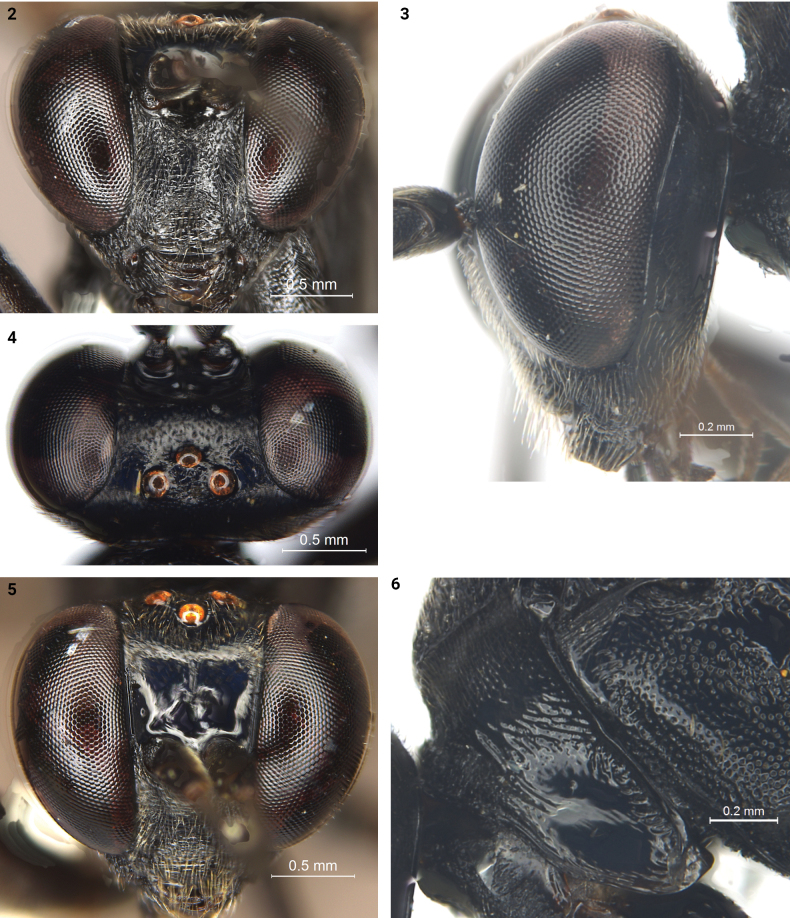
*Trychosisnaolihense* Meng & Ren.: **2** holotype, female, head, anterior view **3** holotype, female, head, lateral view **4** holotype, female, head, dorsal view **5** holotype, female, head, dorso-anterior view **6** holotype, female, pronotum and part of mesopleuron, lateral view.

***Head*.** Face (Fig. 2) 1.2× as long as wide, slightly convex medially; lateral margin almost parallel; dorsal portion with dense fine punctures, ventral portion with indistinct, short, transverse wrinkles; dorsal margin evenly concave medially. Clypeus (Fig. 2) 1.2× as wide as long, with dense, weak, transverse wrinkles, and fine, sparse, indistinct punctures laterally; subventral margin depressed. Mandibles with dense, obscure rugae; dorsal tooth as long as ventral tooth. Malar space rough, 0.8× as long as basal width of mandible. Gena (Figs 3, 4) strongly convergent in dorsal view, in lateral view approximately 0.4× as long as width of eye, with dense fine punctures. Vertex (Fig. 4) with indistinct fine punctures, denser on posteromedian part. Stemmaticum slightly convex, irregularly rugulose. Postocellar line approximately 1.3× as long as ocular–ocellar line. Frons with median longitudinal carina (Fig. 5), dorsal part with dense, indistinct punctures, ventral part smooth, shiny. Antenna stout, with 23 flagellomeres; ratio of length from the first to the fifth flagellomeres approximately: 6.1: 6.4: 5.7: 4.8: 4.5. The first flagellomere 4.5× as long as its maximum width. Occipital carina complete and strong, with the ventral end joining the hypostomal carina slightly posterior to the base of mandible.

**Figures 7–12.**
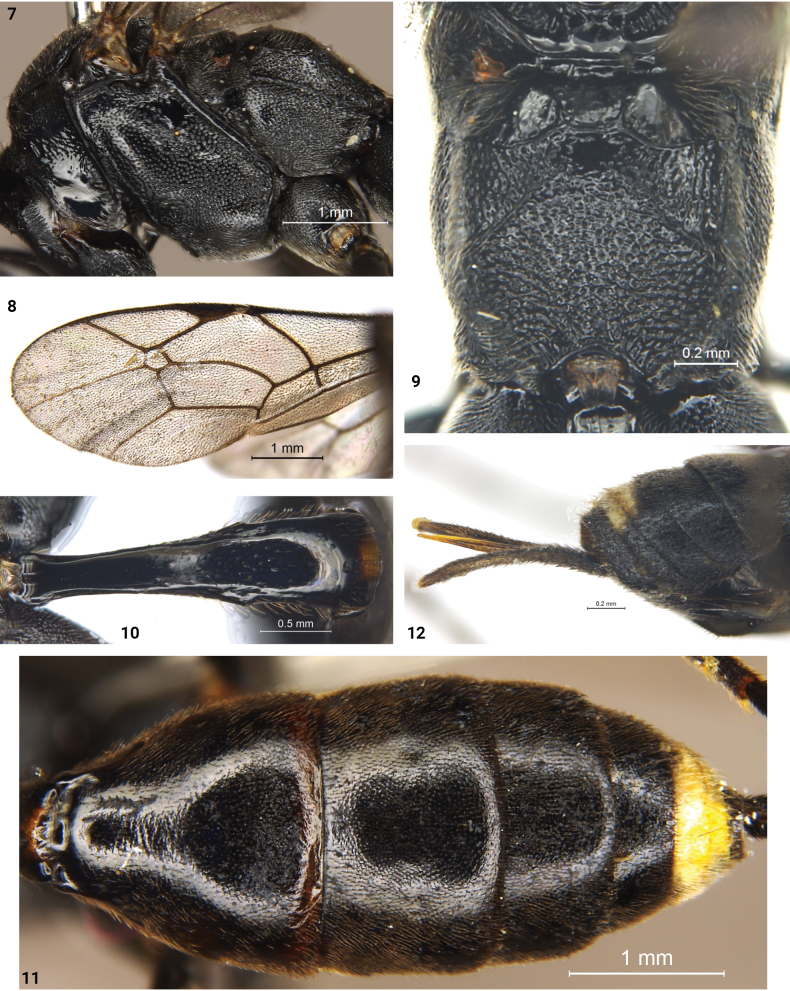
*Trychosisnaolihense* Meng & Ren. Holotype, female, mesosoma, lateral view **8** holotype, female, fore wing **9** holotype, female, propodeum **10** holotype, female, abdominal tergite 1, dorsal view **11** holotype, female, abdominal tergites to 8, dorsal view **12** holotype, female, posterior portion of metasoma, lateral view.

***Mesosoma*.** Lower part of propleuron (Fig. 6) smooth and shiny in lateral view, with short, transverse wrinkles along ventral posterior margin; pronotum with oblique wrinkles medially, with dense, fine punctures posteriorly in dorsal view. Epomia present and short. Mesoscutum slightly convex and weakly punctate, anterior part of notauli present. Scutellum evenly convex, with dense, fine punctures, laterally with indistinct, short, longitudinal wrinkles; anterior part 0.2 with lateral carina. Postscutellum shiny, transverse posteriorly, anterior part concave, laterally with deep pit. Mesopleuron (Fig. 6) with dense punctures; distance between punctures 0.2–2.5× diameter of a puncture. Speculum small, smooth. Upper end of epicnemial carina reaching mid part of pronotum, distant to anterior margin of mesopleuron. Mesosternum with dense fine punctures, and short indistinct wrinkles near epicnemial carina. Metapleuron (Fig. 7) with dense punctures; distance between punctures 0.2–0.5× diameter of a puncture; ventral posterior part with oblique transverse wrinkles. Juxtacoxal carina absent. Posterior spur of hind tibia longer than anterior spur, reaching to the middle of the first tarsomere. Ratio of length of hind tarsomeres from the first to the fifth approximately: 9.0: 3.5: 2.4: 1.7: 2.3. Wings (Figs 1, 8) slightly gray, hyaline. Fore wing with vein 1cu-a almost interstitial M&RS. Areolet pentagonal, convergent anteriorly, receiving vein 2m-cu in the middle, 3rs-m shorter than 2rs-m. 2m-cu distinctly reclivous. Postnervulus intercepted at about lower 1/4. Hind wing vein 1-cu 1.9× as long as cu-a. Propodeum (Fig. 9) with anterior carina complete and strong; posterior carina present laterally; the anterior part between the anterior margin and the anterior carina shiny and almost smooth; posterior part rough, with dense, indistinct, fine punctures. Propodeal spiracle small, transversely elliptical.

***Metasoma*.** Tergite 1 (Fig. 10) approximately 3.9× as long as posterior wide. Petiole smooth dorsally, almost parallel laterally; lateral sides with dense, indistinct, short, longitudinal wrinkles. Postpetiole slightly widened posteriorly, 1.4× as long as the maximum width, with antero-median part with few fine punctures, without latero-median carina, and with dorso-lateral carina incomplete and weak. Spiracle small, circular, located at posterior 0.4 of the first tergite. Tergite 2 (Fig. 11) strongly widened posteriorly, approximately as long as posteriorly wide, finely shagreened, with transverse, fine, indistinct wrinkles at anterior median portion. Tergite 3 (Fig. 11) with sculpture as on tergite 2, almost parallel laterally, 0.6× as long as posteriorly wide. Ovipositor sheath approximately 5.5× as long as hind tibia. Ovipositor (Fig. 12) elongate, evenly and sharply pointed apically.

#### Coloration

**(Fig. 1).** Body almost entirely black, except tergites 6–7 yellowish white dorsally. Antenna concolorous with body. Legs black with basal 0.7 of hind femur red. Wings slightly infumated; pterostigma and wing veins brownish black.

#### Etymology.

The specific name is derived from the type locality.

#### Distribution.

Heilongjiang, China.

#### Remarks.

The new species is similar to *Trychosisnigra* (Telenga, 1930) in having gena that almost straightly converge backwards (Figs 3, 4); anterior part of scutellum with lateral carina; anterior part of propodeum between anterior margin and anterior carina smooth and shiny; head, mesosoma, and tergites 1–5 entirely black. The female of this new species can be distinguished from *T.nigra* by the combination of the following characters: fore wing with vein 1cu-a interstitial with vein M&RS, the maximum width of areolet at most equal to its height, hind wing with vein 1-cu distinctly longer than cu-a; legs entirely black except for basal 0.75 of the hind femur red; tergites 5–6 yellowish white dorsally. Whereas the females of *T.nigra* have the fore wing with vein 1cu-a distinctly postfurcal to M&RS; width of areolet distinctly wider than its height, hind wing vein 1-cu shorter than cu-a; the fore and middle legs with the femora apically and the tibiae entirely red, the hind leg with the tibia basally red and the femur entirely black; tergites entirely black.

## Supplementary Material

XML Treatment for Trychosis

XML Treatment for Trychosis naolihense

## ﻿Additional information

### Author ORCIDs

Ying-Jie Ren https://orcid.org/0000-0002-8472-8992

### Data availability

All of the data that support the findings of this study are available in the main text or Supplementary Information.
